# Transition to language: From agent perception to event representation

**DOI:** 10.1002/wcs.1594

**Published:** 2022-05-31

**Authors:** Klaus Zuberbühler, Balthasar Bickel

**Affiliations:** ^1^ Institute of Biology, University of Neuchatel Neuchatel; ^2^ School of Psychology and Neuroscience University of St Andrews St Andrews; ^3^ Department of Comparative Language Science University of Zurich Zurich Switzerland; ^4^ Center for the Interdisciplinary Study of Language Evolution University of Zurich Zurich Switzerland

**Keywords:** event perception, evolution of grammar, script theory, theory of mind, vocal learning

## Abstract

Spoken language, as we have it, requires specific capacities—at its most basic advanced vocal control and complex social cognition. In humans, vocal control is the basis for speech, achieved through coordinated interactions of larynx activity and rapid changes in vocal tract configurations. Most likely, speech evolved in response to early humans perceiving reality in increasingly complex ways, to the effect that primate‐like signaling became unsustainable as a sole communication device. However, in what ways did and do humans see the world in more complex ways compared to other species? Although animal signals can refer to external events, in contrast to humans, they usually refer to the agents only, sometimes in compositional ways, but never together with patients. It may be difficult for animals to comprehend events as part of larger social scripts, with antecedent causes and future consequences, which are more typically tie the patient into the event. Human brain enlargement over the last million years probably has provided the cognitive resources to represent social interactions as part of bigger social scripts, which enabled humans to go beyond an agent‐focus to refer to agent–patient relations, the likely foundation for the evolution of grammar.

This article is categorized under:

Cognitive Biology > Evolutionary Roots of Cognition
Linguistics > Evolution of Language
Psychology > Comparative

## Graphical abstract

Animals and humans may perceive events in similar ways but most likely process them differently in line with their respective representational abilities, ranging from agent‐biased actions, anchored in the present, to agent‐patient social scripts that extend into the future.

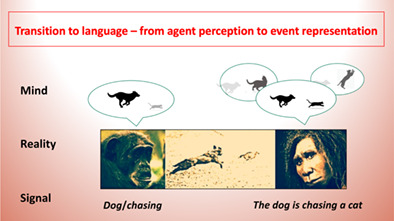

## 1 HALLMARKS OF LANGUAGE

Humans are unique in a number of ways but perhaps most strikingly in their capacity for language. No other animal species has a communication system even remotely comparable, a fact that has puzzled scholars for centuries (Darwin, 1871). How could a complex faculty, such as language, have evolved during the relatively short evolutionary history of our species? One way of studying the problem is to decompose language into core properties and investigate the associated capacities separately (Hurford, 2007). To this end, studying the communication systems of primates and other animals has been particularly enlightening. The approach is based on the premise that evolutionary adaptations are often modifications of preexisting functions rather than true innovations. While this approach has revealed continuities between animal and human communication, particularly regarding the attribution of meaning, it has also highlighted ways by which humans deviate substantially from what is normally observed in primate communication, specifically vocal control and social cognition (W. T. Fitch & Zuberbühler, 2013).

## 2 EVOLUTION OF VOCAL CONTROL

By default, language is strongly tied to the acoustic mode. From early on infants begin to playfully babble with sounds, a behavior not observed in other primate species (Oller et al., 2013). For instance, chimpanzee infants remain mainly silent during their first years (Laporte & Zuberbuehler, 2011), babbling is a rare exception in primate vocal development (Elowson et al., 1998) and as adults primates continue to have limited vocal control and no real capacity for vocal learning, even with substantial training (Cheney & Seyfarth, 2005). It has also been noted that great apes are not interested in imitating human speech although, in other contexts, they like copying human behavior. Hayes and Hayes (1951) write about their home‐raised chimpanzee “Viki”:

… Just as the human child copies its parents' routine chores, so Viki dusts, washes dishes, sharpens pencils, saws, hammers … […]. On the other hand, she is less vocal: while the human child commonly keeps up an almost continual stream of chatter—with or without meaning, Viki is silent.

Why are such seemingly trivial vocal imitation tasks near impossible for primates? One popular explanation has been to invoke anatomical differences of the vocal tract, particularly the permanently lowered human larynx (Lieberman, 2012). But this view is no longer supported by current research. The primate vocal tract, as it has been put, is perfectly speech‐ready, despite the fact that speech has only evolved in humans (W. Fitch et al., 2016). Also relevant is that great apes are capable of controlling the supra‐laryngeal vocal tract to produce various voiceless signals, such as clicks, smacks, raspberries, kiss‐sounds, and whistles (Lameira et al., 2014), suggesting that key articulatory movements have evolved before speech. Furness (1916) wrote of his home‐raised orangutan:

The orang in one respect does use the lips, to make a sound indicating warning or apprehension; this sound is made with the lips pursed up and the air sucked through them … […] My oldest orang would make this sound on command (I had merely to say “What is the funny sound you make when you are frightened?”).

Producing sustained airflow to activate the vocal folds, however, appears to be much harder for nonhuman primates. Related, it is difficult to train primates to vocalize on command, suggesting that control over the larynx and respiratory muscles may have evolved more recently. On this, Hayes and Hayes (1951) write:

The first step was aimed at teaching her merely to vocalize on command, in order to obtain a reward. […] The task was surprisingly difficult. Although she seemed to learn what was required quickly, she had serious trouble with the motor skill of voluntary vocalization.

This is remarkable because vocal learning appears to evolve relatively easily across the animal kingdom (Vernes et al., 2021) although usually for functions related to reproduction or social cohesion. Examples are birdsong (Doupe & Kuhl, 1999) and or the song of cetaceans (Janik, 2009), dolphin whistle imitations (Janik, 2000) or vocal convergence in primate social groups, usually as a means to signal social closeness (Ruch et al., 2018). Human speech, on the other hand, does not just function in sexual rivalry or as social markers; it is a semantic device allowing speakers to refer to reality the way they perceive or remember it.

One hypothesis is that the transition to vocal control in humans occurred in the context of cooperative breeding (Hrdy, 2009; Zuberbühler, 2011). Humans are unusual in the amount of childcare they provide, both in traditional hunter‐gatherer and in modern societies. Often, this involves unrelated individuals, and this may especially be challenging for infants. Advanced vocal control, beyond a basic primate‐like call repertoire, may have evolved to help infants to secure care from older individuals who often do not have a genetic interest to do so. In particular, natural selection may have favored vocal behavior in early human infants that facilitated attention getting and social bonding, especially with unrelated individuals. Babbling may be particularly important (Darwin, 1871), if it helps human infants to be noted by caregivers and to facilitate social bonding with them. Interestingly, babbling is generally perceived as “pleasant,” suggesting that natural selection may have benefitted from a preexisting receiver bias. The hypothesis that babbling elicits infant care more efficiently than other vocal behavior requires further testing, ideally across different primate species.

Another hypothesis is that vocal control evolved in response to the demands of higher social cognition. Human evolution is characterized by a considerable brain enlargement starting around 1 mya (Ponce de León et al., 2021), which must have led to more complex representational abilities, especially in the social domain (Dunbar & Shultz, 2007). As humans were able to mentally represent social information in more complex ways, this may have created selection pressure on the communication system. Increased vocal control may have been an evolutionarily easy solution, endowing early humans with an ability to create a wider range of utterances to refer to the world than with a rigid signal repertoire. The hypothesis that vocal control may have evolutionarily followed advanced representational abilities is in line with research on sign language, which has shown that language does not require speech.

## 3 FROM COMBINATORIAL TO COMPOSITIONAL

Animal communication can be meaningful at various levels. First, if an individual consistently produces the same signal to a specific event, the signal becomes a predictor, a relation animals understand quickly by associative learning. But meaning is sometimes conveyed in more complicated ways, for example, via the context of signal production. For example, Guinea fowls in Tai Forest alarm call to both humans and leopards. Diana monkeys appear to be aware of this as they respond differently to the birds' alarm calls depending on whether they suspect humans or leopards to be the cause of the calls (Zuberbuhler, 2000). Extracting meaning from taking context into account may in fact be the default mechanism in animal communication (R. Seyfarth & Cheney, 2018; Smith, 1977). Here, there are interesting links to pragmatic abilities in humans, although these go beyond what is normally seen in animal communication, mainly due to the fact that they involve generalised reasoning about intentions (Levinson, 2000). Although great apes (and probably other animals) can assess others' intentions during communication (e.g., Genty et al., 2015), they do not appear to make active use of their own intentions during signal production to generate pragmatic meaning.

Finally, meaning in animal communication can also be transmitted by signal combinations. Examples are unordered sequences where meaning resides in the distribution of bigrams (e.g., duplications): in titi monkeys, listeners react to the proportion of one type of bigram relative to others, allowing them to infer the type and location of danger (Berthet et al., 2019). Similarly, bonobos produce call sequences with bark or peep bigrams to preferred foods and peep‐yelp and yelp bigrams to non‐preferred foods (Zuberbühler, 2020). In playback experiments listener responded in appropriate ways, suggesting they managed to extract meaning from these sequences (Clay & Zuberbühler, 2011).

Sometimes, signal sequences have identifiable orders (i.e., permutations). This has been reported, for example, in Diana monkey contact calls (Coye et al., 2018), Campbell's monkey alarm call suffixations, Campbell's monkey boom/alarm combinations (Ouattara et al., 2009) or putty‐nosed monkey pyow‐hack combinations (Arnold & Zuberbuhler, 2006). In all cases, there is corresponding experimental evidence that listeners attend to the changes in meaning induced by the permutations (Arnold & Zuberbühler, 2008; Coye et al., 2015, 2016; Zuberbuhler, 2002). Great apes also produce permutations (Crockford & Boesch, 2005; Fedurek et al., 2016; Leroux et al., 2021) but it is currently unclear how the meaning of calls and sequences interact.

Per definition, compositionality requires that the meaning of a complex expression depends on the meaning of its parts and the way they are combined, that is, the meanings of the composing units and the way they related to one another (Johnson, 2021). Campbell's monkey suffixation is an interesting example, because the ‐oo modulation, given to non‐dangerous eagle or leopard‐related situations, operates like an affix in human languages (e.g., the plural ending ‐s for English nouns) (Ouattara et al., 2009). Affixes are meaningful but they cannot occur on their own and bear a specific meaning relation to their host. There are also good candidates for compositionality from other groups of animals, especially in birds (Engesser et al., 2016; Suzuki et al., 2016), although there are alternative explanations (Arnold & Zuberbühler, 2012; Schlenker et al., 2016) and animal compositionality might be restricted to simple “and” relations (Townsend et al., 2018).

## 4 FROM AGENTS TO EVENTS

One key area where human languages exploit compositionality is in event coding. Many sentences consist of arguments that describe how agents as causal forces bring about states of affairs to patients. There is a strong trend in languages to have explicit grammatical signals that allow listeners to distinguish the agent from a patient and the nature of their interaction. English uses word order, other languages use special markers. Across languages, agents are perceived as privileged insofar as they exert physical or psychological power over patients or states of affairs (Sauppe & Flecken, 2021; Dowty, 1991).

Do animals perceive the world in the same way, that is, as causally structured agent and patient interactions? One source of information comes from ape language studies (Tomasello, 2017). Here, apes have demonstrated that they can understand their human caretakers to an astonishing degree, including instructions that contain agent–patient relations (Savage‐Rumbaugh et al., 1993). At the same time, however, there was no compelling evidence that any of the language‐trained apes were capable of constructing basic sentences with agent–patient interactions. Research on natural animal communication shows the same pattern. On the one hand, playback experiments have shown that monkeys and apes can make basic inferences about who‐does‐what‐to‐whom, by simply listening to call exchanges between two individuals (Bergman et al., 2003; Slocombe et al., 2010). But again, in terms of production, there are no good examples from any species indicating that animals can refer to the agent–patient relations of an event (Wilson et al., 2022; Zuberbühler, 2021). Hence, the tentative current conclusion is that animals understand social interactions in terms of agency, but are unable to convey this in their signal production, in line with previously reported signaller–receiver differences in animal communication (R. M. Seyfarth & Cheney, 2003). The currently known cases of compositionality in animal communication refer to either the agent, for instance by producing acoustically distinct suffixed or unsuffixed alarm calls that specify not only the predator type but also its current level of threat (Ouattara et al., 2009), or to desired responses by recipients (Engesser et al., 2016) with no reference to patients. Animal vocalisations are often self‐reports which denote agents or patients, but never both (e.g., “I am digging”, “I am moving”: Jansen et al., 2012). Nonhuman primates, as it has been eloquently put, are “dendrophobic” (unable to mentally represent or manipulate tree structures) whereas human infants gradually become dendrophilic during their linguistic development (Trueswell, 2017).

Why are humans prone to integrate patients with agents into hierarchical structures, at least in their communication? Natural events are embedded in time, with past histories and future consequences. If an animal witnesses an event (e.g., A bites B) then this is most likely a consequence of a prior event (e.g., B threatened A's infant) and will likely have future consequences (e.g., B avoids A). Human event cognition may be more complex insofar as events are not perceived as stand‐alone experiences, but as embedded in time with a past and future. Perceiving events in time, according to this hypothesis, gives patients shadow agency as active participants (either in the past or future); they participate in larger scripts of how social interactions unfold with constantly changing agency (Fillmore, 1982). Humans, when witnessing an agent acting upon a patient, may already see the patient as the future agent. If this is true then grammar may only evolve in minds that can represent the world beyond the here‐and‐now, with its natural focus on agents. Related to this and in analogy to the main sentence functions of human language (i.e., declarative, interrogative, exclamative, and imperative), animal communication appears to be restricted to the exclamative and imperative, that is, functions in which the attention is on the present state and the agent.

## 5 FROM EVENTS TO SCRIPTS

Recently, we have argued that humans (and animals to various degrees) solve social problems by activating behavior scripts, accumulated with ontogenetic experience and phylogenetic predisposition (Taylor et al., under review; Zuberbühler, 2022). Behavior scripts are patterns of how interactions typically unfold in reality and are stored in memory as such. Behavior scripts have a temporal dimension with entry points, allowing subjects to recognize and categorize ongoing scripts from incomplete event input, and make predictions about what will happen next. We also argued that behavior script theory can explain subjects' performance in knowledge‐ignorance paradigms (false‐belief tasks), such the Sally–Anne test (human infants) (Baron‐Cohen et al., 1985), the snake paradigm (wild chimpanzees) (Crockford et al., 2012) or the King Kong paradigm (captive apes) (Krupenye et al., 2016). Briefly, in the Sally–Anne test, children see two puppets interacting. The gist of the story is that Anne removes Sally's marble in her absence. Children then have to predict where Sally, upon her return, will look for her marble. From around 4 years, children say that Sally will search where she left her marble (old location), while younger children say that Sally will search where Anne moved the marble (new location), suggesting that attributing false beliefs is part of cognitive development. However, it is conceivable that children solve the Sally–Anne test by reference to a simple behavior script, for example, that “people return to where they have left stuff,” something they may learn from own experience or from observing others. Similar behavior‐based arguments can be made for false‐belief experiments with apes (Taylor et al., under review). Complex theory‐of‐mind based notions, such as “Anne has a false belief” may be linguistic shortcuts to refer to situations where two behavior scripts are in conflict and require a decision (Sally goes to the marble; Sally goes to where she left the marble). The fact that only 4‐year olds respond correctly may simply reflect the fact that, at a younger age, children do not have enough experience to understand that current events can be explained by past events.

Indeed, scripts (or “frames”) are well‐known to be the critical basis for semantics and grammar in language (Fillmore, 1982). They are an essential part of the evolutionary package that languages endow humans with. The depth of event perception, in other words, may depend on an individual's resources and experience, both cognitively and linguistically. The key variable is how much an individual can deviate from the present and recognize events as part of larger scripts of event sequences that are logically connected and unfold according to basic laws of social behavior. The more restricted to the present, the more individuals will focus on agents. With increasing perception of time, the same agent–patient interaction will be increasingly perceived as part of larger script. Perceiving the world in this way will turn attention to the patients of ongoing events and in the corresponding signal output.

## 6 CONCLUSIONS

How did human communication transition from primate‐like vocal behavior to grammatically organized spoken language? The argument is that speech was made possible by granting a speech‐ready vocal apparatus voluntary control over sound production, in concert with already existing articulatory control. This was likely an evolutionary response to early humans perceiving social interactions in increasingly complex ways, with corresponding demands on the communication system. This may have been in terms of perceiving events beyond their present appearance, to include a timeline of prior causes and future consequences. Adding such a temporal dimension to event perception will reduce the strong agent focus that appears to dominate animal cognition, in favor of more balanced agent–patient perception. Humans may be the only species capable of integrating reference to a patient with reference to an agent, thus removing a major obstacle in the evolution of grammar.

## AUTHOR CONTRIBUTIONS

**Klaus Zuberbühler:** Conceptualisation (equal); funding acquisition (equal); Writing – original draft. **Balthasar Bickel:** Conceptualisation (equal); funding acquisition (equal); Writing – review and editing.

## CONFLICT OF INTEREST

The author have declared no conflicts of interest for this article.

## RELATED WIREs ARTICLES

Events parsing and the origins of grammar

Linguistic capacity of non‐human animals

## Data Availability

Data sharing is not applicable to this article as no new data were created or analyzed in this study.
